# Differential production rates of cytosolic and transmembrane GFP reporters in *C. elegans* L3 larval uterine cells

**DOI:** 10.17912/micropub.biology.000813

**Published:** 2023-03-21

**Authors:** Isabel Kenny-Ganzert, Qiuyi Chi, David Sherwood

**Affiliations:** 1 Department of Biology, Duke University

## Abstract

Transgene driven protein expression is an important tool for investigating developmental mechanisms in *C. elegans* . Here, we have assessed protein production rates and levels in L3 larval uterine cells (UCs). Using ubiquitous promoter driven cytosolic and transmembrane tethered GFP, fluorescence recovery after photobleaching, and quantitative fluorescence analysis, we reveal that cytosolic GFP is produced at an ~two-fold higher rate than transmembrane tethered GFP and accumulates at ~five-fold higher levels in UCs. We also provide evidence that cytosolic GFP in the anchor cell, a specialized UC that mediates uterine-vulval connection, is more rapidly degraded through an autophagy-independent mechanism.

**Figure 1. Cytosolic GFP accumulates at higher levels and is produced more rapidly than transmembrane tethered GFP in mid L3 larval uterine cells (UCs).**
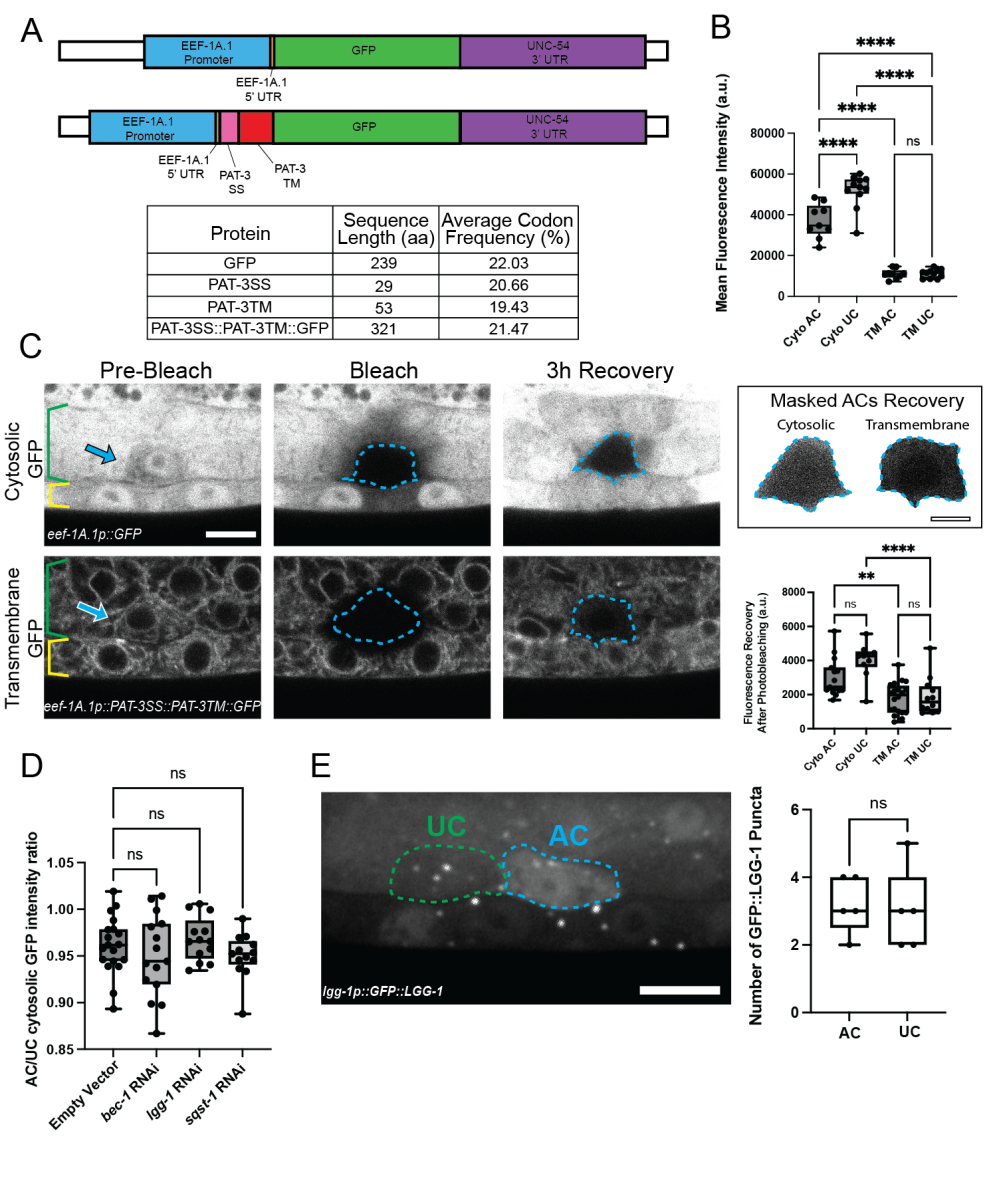
**(A)** A schematic diagram of constructs for cytosolic GFP and the transmembrane tethered GFP generated using the PAT-3 (b integrin subunit) signal sequence (PAT-3SS) and PAT-3 transmembrane domain (PAT-3TM). The PAT-3 transmembrane domain retains the protein in the secretory apparatus, allowing assessment of protein production as the GFP signal is easily quantified within the secretory apparatus of individual cells. Both constructs were driven by the ubiquitous promoter *eef-1A.1* . Table shows amino acid sequence length and average codon frequency of the codons used to encode the protein or protein region indicated (see Methods). **(B)** Graph shows cytosolic (Cyto) and transmembrane (TM) tethered GFP fluorescence levels in the anchor cell (AC) and neighboring UCs (ventral UCs, see Methods). n > 10, **** p < 0.0001, ns (not significant) p > 0.05, one-way ANOVA, Tukey’s multiple comparison test. **(C)** (Left) Fluorescence micrographs show the AC (arrow) and UCs (green brackets) over the vulval precursor cells (yellow brackets) prior to photobleaching the AC (Pre-Bleach), right after photobleaching (blue dashed line, Bleach), and then after 3 hours (h) of recovery (3 h Recovery). Note that prior to photobleaching, the levels of cytosolic GFP are lower in the AC compared to the uniform high expression in other UCs and that transmembrane tethered GFP levels are uniform in all UCs (n > 50/50 observed for each). Bar = 5µm. (Right) Masking of the AC after 3 h of recovery highlights differences in GFP recovery after photobleaching. Bar = 2.5µm. Graph displays GFP recovery rates. n > 10, **** p < 0.0001, ** p < 0.01, ns p > 0.05, one-way ANOVA, Tukey’s multiple comparisons test. **(D)** AC/neighboring UC cytosolic GFP ratio after control RNAi (empty vector) or RNAi treatment against autophagy genes. n > 13, ns p > 0.05, one-way ANOVA, Dunnett’s multiple comparisons test. **(E)** The autophagosome marker GFP::LGG-1 driven by its own promoter. GFP::LGG-1 puncta mark autophagosomes in the AC (blue dashed line) and neighboring UC (green dashed line). Bar = 5µm. Graph displays number of GFP::LGG-1 puncta. n = 5 each, ns p > 0.05, Student’s *t* test.

## Description

The developing *C. elegans* uterus is an important model for investigating mechanisms underlying cell fate, cell proliferation, morphogenesis, transorganogenesis, and necrosis (Cinar et al., 2003; Dobrzynska & Askjaer, 2016; Ghosh & Sternberg, 2014; McClatchey et al., 2016; Newman et al., 1996; Reza et al., 2022; Riddle et al., 2016). Studies on the anchor cell (AC), a specialized uterine cell (UC) that mediates the uterine-vulval connection (Sherwood & Plastino, 2018), have also provided insight into transcriptional regulation, cell invasion, cell-cell fusion, and lateral and inductive signaling (Attner et al., 2019; Hill & Sternberg, 1992; Sapir et al., 2007; Sherwood & Sternberg, 2003; Spiri et al., 2022; Wilkinson et al., 1994). A key tool for experimentally examining *C. elegans* developmental mechanisms are cell-type specific and ubiquitous promoters that drive protein expression for rescue, protein misexpression, fluorophore transcriptional reporters and molecular sensors (Adikes et al., 2020; Garde et al., 2022; Sherwood et al., 2005). The general efficiency of cytosolic and transmembrane protein production and accumulation of proteins in UCs, however, has not been examined.

To better understand how proteins are produced in the AC and neighboring UCs during the mid L3 larval stage of development, we examined transgenic worms expressing cytosolic GFP and a transmembrane tethered GFP ( PAT-3 SS:: PAT-3 TM::GFP) under the ubiquitous *eef-1A.1* promoter ( **Figure 1A** ) (Tomioka et al., 2016). We used the transmembrane domain of PAT-3 , as it is retained in the secretory apparatus of UCs (Hagedorn et al., 2009) and allows clear assessment transmembrane tethered GFP produced within individual UCs. To ensure similar transgene expression, we used Mos1-mediated Single Copy Insertion (MosSCI) to insert a single copy of each transgene into the same site on Chromosome I (Frokjaer-Jensen et al., 2012; Philip et al., 2019). We found ~4-5 fold higher levels of cytosolic GFP expression in the AC and neighboring ventral UCs compared to levels of the transmembrane tethered GFP ( **Figure 1B,C** ), suggesting that the transmembrane tethered GFP might not be produced as efficiently. To assess production rate, we conducted fluorescence recovery after photobleaching (FRAP) experiments. Notably, there was ~two-fold higher levels of cytosolic GFP in the AC and neighboring UCs compared to the transmembrane tethered GFP in the AC and UCs 3 hours after photobleaching, indicating more rapid production of cytosolic GFP. The only difference between the cytosolic and transmembrane tethered GFP constructs was the presence of the transmembrane domain and signal sequence in PAT-3 SS:: PAT-3 TM::GFP, which added 82 amino acids in length onto the 239 amino acid GFP. The transmembrane domain and signal sequence also had a similar average codon frequency to GFP ( **Figure 1A** ) (Nakamura et al., 2000). This suggests that the presence of rare codons and the slightly increased size of the transmembrane tethered GFP are likely not significantly slowing protein production (Clarke & Clark, 2008). Other mechanisms, such as signal recognition particle pausing of translation prior to endoplasmic reticulum docking for cotranslational translocation, might instead reduce the speed of the transmembrane tethered GFP production (Mahlab & Linial, 2014).

Another difference in protein levels was evident from observing cytosolic GFP. Although FRAP data suggested that cytosolic GFP production rate in the AC was the same as in neighboring ventral UCs, the overall levels of the cytosolic GFP in the AC was less than the neighboring UCs, where it appeared uniformly higher ( **Figure 1B** , **C** ). This difference was not observed with transmembrane tethered GFP ( **Figure 1B,C** ). This suggests that cytosolic GFP might be degraded at a faster rate in the AC than in neighboring UCs. As autophagy can selectively degrade cytosolic proteins through bulk removal (Aman et al., 2021), we examined the effects of RNAi mediated reduction of the key autophagy regulatory genes *bec-1* , *lgg-1 ,* and *sqst-1* (Chen et al., 2017) on the levels of cytosolic GFP in the AC relative to neighboring UCs. Cytosolic GFP levels, however, were unchanged after all RNAi treatments ( **Figure 1D** ). While these findings are consistent with autophagy not being responsible for reduction of cytosolic GFP in the AC, a caveat of this experiment is that we did not confirm RNAi-mediated reduction of the protein products of *bec-1* , *lgg-1 ,* and *sqst-1 .* We also examined the autophagosome marker GFP::LGG-1 driven by its own promoter (Lapierre et al., 2013). We note that there was more diffuse cytosolic GFP::LGG-1 in the AC versus neighboring UCs (n = 5/5 observed), a phenomenon that has been observed in other cells with this marker (Chen et al., 2017). Importantly, no difference was detected in the prevalence of GFP::LGG-1 puncta, which mark autophagosomes (Chen et al., 2017; Lapierre et al., 2013) ( **Figure 1E** ). In sum, our results indicate that cytosolic GFP is produced at a higher rate and accumulates at greater levels in UCs than a transmembrane tethered GFP. Our studies further suggest that the AC might have a higher general rate of cytosolic protein degradation through an autophagy-independent mechanism. These findings may apply to other cytosolic and transmembrane proteins expressed in developing cells in *C. elegans* and should be considered in future studies.

## Methods

**Strain maintenance:**

*Caenorhabditis elegans* were grown under standard conditions on nematode growth media (NGM) seeded with OP50 *Escherichia coli* at 20°C (Stiernagle, 2006). Animals were synchronized using a bleaching treatment of gravid adults to isolate embryos, which were then grown in M9 media without food to arrest development at the L1 stage (Porta-de-la-Riva et al., 2012). The genotypes of all strains used in this study are shown in Strain Table.

**Transmembrane GFP construct:**

To generate the *eef-1A.1p* :: *pat-3 ss:: pat-3 tm::* GFP construct, Apal was used to cut a multiple cloning site vector pCFJ352 that contained *eef-1A.1p* ::GFP (Plasmid pQD01). Primers were used to amplify the signal sequence (SS) and transmembrane domain (TM) encoded by *pat-3* gene (the sole *C. elegans* b integrin chain) from Fire Vector pPD122.39. The Fire Lab C. elegans Vector Kit was a gift from Andrew Fire (Addgene kit # 1000000001). The *pat-3* sequence encoding the SS and TM were inserted between the *eef-1A.1* promoter and GFP using Gibson Assembly. CRISPR/Cas9-mediated genome editing was then used to insert *eef-1A.1 p:: pat-3 ss:: pat-3 tm::GFP* into the standard MosSCI insertion site ttTi4348 on Chromosome I (Frokjaer-Jensen et al., 2012; Philip et al., 2019).

**Calculation of average codon frequency:**

A table of codon frequencies for *C. elegans* was obtained from the Codon Usage Database ( http://www.kazusa.or.jp/codon/ (Nakamura et al., 2000)). The coding sequences for GFP, *pat-3 ss* , and *pat-3 tm* were split into codons using ApE – A plasmid Editor v2.0. The frequency for each codon encoding GFP, PAT-3 SS and PAT-3 TM was then determined, summed, and then averaged to determine average codon frequency percentage.

**RNA interference (RNAi):**

All RNAi constructs were obtained from the ORF-RNAi V1.1 library *elegans* (Open BioScience) (Rual et al., 2004). RNAi knockdown was performed using the feeding method in the HT115 *E. coli* strain (Timmons et al., 2001) according to previously described protocols (Costa et al., 2023). Briefly, synchronized L1 larvae were plated on RNAi plates, grown at 20°C for 36-40 hours until the L3 stage, and then imaged. The HT115 *E. coli* containing empty L4440 vector was used as a negative control.

**Image acquisition** :

Images were acquired on an inverted Zeiss 880 point scanning confocal mounted on a Zeiss Axio Observer Z1 microscope and 63x 1.4 NA oil immersion objective. For all images the pinhole size was set to 1 A.U. and GFP was excited with 488 nm laser and collected with GaAsP detector set to 498-553 nm range. Animals were anesthetized by soaking in 5 mM Levamisole in M9 for 15 minutes then transferred to a 5% noble agar pad. A cover slip was place on top of the worms and sealed with VALAP to prevent the slide from drying out and flooded with 5 mM Levamisole to provide prolonged anesthetizing (Kelley et al., 2017). The free hand ROI tool was used to circle the AC or UC, then 15 iterations of simultaneous 405 nm (100% laser power) and 488 nm (100% laser power) was used to photobleach fluorescence signal within the AC or UC. Worms were imaged prior to and immediately following bleaching, then again 3 h later.

Images of cytosolic GFP after RNAi targeting autophagy regulatory genes were acquired on a Zeiss Axio Imager Microscope equipped with a Yokogawa CSU-10 spinning disk using a 100x Plan-APOCHROMAT 1.4NA oil immersion objective and Hamamatsu Orca-Fusion sCMOS camera. Images of GFP::LGG-1 puncta were acquired on a Zeiss Axio Imager Microscope equipped with a Yokogawa CSU-10 spinning disk using a 100x Plan-APOCHROMAT 1.4NA oil immersion objective and ImageEM EMCCD camera without gain. For both the autophagy RNAi and GFP::LGG-1 experiments, worms were mounted on 5% noble agar pads containing 0.01 M sodium azide.

**Image analysis:**

All image quantification was performed in Fiji 1.53f (Schneider et al., 2012). Imaging parameters were identical in experiments so fluorescence intensity could be compared. Anchor cell (AC) or an adjacent ventral uterine cell (UC) mean fluorescence intensity was determined using the free hand tool in Fiji to circle the cells to determine the average fluorescence/unit area. For FRAP experiments, a bleach correction factor was calculated to account for general photobleaching (Gianakas et al., 2023). Specifically, a background-corrected fluorescence intensity measurement in the ventral uterine tissue, distant from the AC or ventral UC where FRAP was performed, was measured pre-FRAP and at the 3 h recovery time point. This recovery measurement was divided by the pre-FRAP measurement to obtain the bleach correction factor. The bleach correction factor was used to normalize the fluorescence intensities in the region where the FRAP was performed. To calculate the amount of fluorescence recovery, the mean fluorescence intensity of the AC or UC immediately following bleaching was subtracted from the mean fluorescence intensity of the AC or UC after 3 h of recovery to account for any signal remaining immediately after photobleaching. To quantify the number of LGG-1::GFP puncta, the AC or UC was circled and bright, in focus puncta were counted by hand.

**Statistical Analysis:**

Data was assessed for Gaussian distribution using a D’Agostino-Pearson omnibus normality test. For normally distributed datasets, an unpaired t-test with Welch’s correction or ordinary one-way ANOVA followed by either Dunnett’s multiple comparisons test or Tukey’s multiple comparisons test was used to determine statistical significance. For datasets that were not normally distributed, a Kruskal-Wallis test followed by Dunn’s multiple comparisons test was used. GraphPad Prism (Version 7) was used for statistical analyses and to generate graphs. For all statistical tests, p < 0.05 was significant. Sample sizes and p values are provided in the figure legend.

## Reagents

**Strains:**

**Table:** 

**Strain**	**Genotype**	**Source**
NK2790	* qy121[ eef-1A.1 p::GFP] II *	(Costa et al., 2023)
NK2933	* qy201[eef-1.A.1p:: pat-3 ss:: pat-3 tm::GFP] II; qyIs50 [cdh-3p::mCherry::moesinABD + unc-119 (+)] *	This study
MAH236	sqIs13 [ *lgg-1 p::GFP:: lgg-1 + odr-1p::RFP]) *	(Lapierre et al., 2013)

**Plasmids:**

**Table:** 

**Plasmid**	**Description**	**Source**
pQD01	ttTi4348 -MCS loxN:: *eef-1.A.1p::GFP::unc-54-3’utr* ::loxN	(Costa et al., 2023)
pQD02	ttTi4348 -MCS loxN:: *eef-1.A.1p:: pat-3 ss:: pat-3 tm::GFP::unc-54-3’utr* ::loxN	This study
